# What matters to people with COPD: outputs from Working Together for Change

**DOI:** 10.1038/s41533-019-0124-z

**Published:** 2019-04-12

**Authors:** Frances Early, Matthew Lettis, Sarah-Jane Winders, Jonathan Fuld

**Affiliations:** 1 Cambridge University Hospitals NHS Foundation Trust, Cambridge, UK; 2 University of Cambridge School of Clinical Medicine, Cambridge, UK; 3 HSE South East and University College, Dublin, Ireland

## Abstract

Chronic obstructive pulmonary disease (COPD) has a negative impact on people’s quality of life affecting daily activities and mental and emotional well-being. Healthcare services need to understand what patients want and need. We used a co-production methodology, Working Together for Change, not previously used in a COPD setting to determine what matters to people with COPD. Forty patients took part in one-to-one discussions to identify what was working well, not working well and what was important for the future in terms of their COPD care. The responses were analysed in two one-day co-production workshops involving COPD patients, carers and professionals. The six highest priority themes around what’s not working well were: ‘I don’t think the right hand knows what the left hand is doing’, ‘I can’t get appointments when I want them’, ‘I’m not treated as a person’, ‘I can’t do what I want to do’, ‘I’m anxious and depressed’ and ‘I can’t eat well.’ Professionals gained powerful insights into the difficulties of COPD through their interactions with patients in the workshops. What mattered to patients encompassed meaning, purpose and relationships beyond immediate medical needs and underlines the need for patient-centred holistic approaches to COPD care and support.

## Chronic lung disease care: What matters most to patients

Interviews and workshops exploring patient perceptions of chronic lung disease care suggest a need to incorporate psychological and practical support with clinical management. Patient psychology frequently affects COPD symptoms; for example, patients with similar levels of physical airflow obstruction experience varying levels of breathlessness and ability to function in day-to-day life. Frances Early at the UK’s Cambridge University Hospitals NHS Foundation Trust and co-workers used a discussion methodology called Working Together for Change to examine positive and negative themes in COPD care. They conducted one-to-one interviews with 40 patients from diverse backgrounds and followed up with workshop discussions involving health professionals and patients to explore emerging themes. Patients coveted a holistic, personalized approach to treatment, including practical support to manage relationships, maintain purpose and enhance emotional well-being.

## Introduction

Chronic obstructive pulmonary disease (COPD) is a progressive lung disease and a significant cause of morbidity and mortality around the globe.^1,2^ It is associated with disabling breathlessness and with frequent infections, hospitalisation and contact with healthcare services.

COPD impacts negatively on quality of life, with breathlessness affecting everyday activities including dressing, washing and eating.^3–5^ Breathlessness is a subjective experience and functional performance can vary among people with the same degree of airflow obstruction.^6^ Spontaneous activity levels may be reduced, particularly as the condition progresses^7,8^ and patients may spend less time engaged in personal care and chores and have lower median sleep duration.^9^ There are further detrimental impacts of social isolation, loneliness, embarrassment, loss of independence and fatigue.^10^ More so than other chronic conditions, COPD causes biographical disruption due to time spent managing health and activities of daily living.^11,12^ Affecting mental and emotional well-being, COPD becomes a way of life for many people with severe disease.^13^

Engagement and participation in a variety of activities enhances well-being and is a legitimate focus for interventions to support people in managing their lives with COPD. It is important to people with COPD to maintain social interactions and a sense of normality, freedom and purpose.^5^ Health services, however, are often based on the medical model and may not address social and emotional aspects of patients’ lives. What matters most to patients and to clinicians may not be the same. Boivin et al. found that patients prioritised primary care access, respect and empathy, whereas professionals focussed more on single-disease management.^14^ Increasing importance is now being placed on providing services that patients recognise as appropriate and relevant.^15^ In order to do this healthcare providers need to get to the heart of what people want and need and understand what matters to them.

To address this need we used a co-production process called Working Together for Change (WTfC)^16^ to generate person-centred, qualitative information to inform the commissioning of COPD services. WTfC has been used effectively in social care and mental health settings but was untried in physical health settings.^17^ We used WTfC to understand what mattered to patients in the context of their COPD and to bring patients and professionals together to explore what patients needed and agree the types of services that would address their needs. An evaluation of the process has been described elsewhere.^18^ In this paper we present outputs of the WTfC process focusing on what mattered to patients.

## Results

### Participants

Sixty-two patients were invited to take part in one-to-one discussions. Forty-five (72%) participated (23 male, 22 female), including one traveller and six oxygen-dependent patients. Some refused due to ill health, sick relatives, other priorities or did not give a reason.

Eleven patients attended the workshops (8 male, 3 female), including one oxygen-dependent patient, along with eight carers (1 male, 7 female). Two patients attended only the second workshop. Some were unable or chose not to attend and some were not confident enough to do so. Two members of the Breathe Easy support group and a lay tutor on a self-management support training programme also attended. Forty-two professionals were invited and 17 (40%) attended (15 female, 2 male), 15 at workshop 1 and 16 at workshop 2. Those who could not attend had previous commitments. A breakdown of participants by patient source and professional organisations is shown in Supplementary Table 1.

### Themes generated from the one-to-one statements

The one-to-one discussions generated 338 responses of which 229 were rated as most important and analysed during the workshops. Of the remaining 109 responses 89 were allocated to workshop-generated themes and 20 were listed separately. Results are presented under two main headings: health care and professional support (Table 1) and living with COPD (Table 2). The summary below contains some example responses that illustrate the themes. All the responses that were collected during the one-to-one discussions are presented in supplement 5.

**Table 1:** Healthcare and professional support themes generated from the one-to-one responses with a short description of each theme [numbers of statements contributing to each theme are shown in brackets as (high priority, lower priority) statements]

	What is working well?	What is not working well?	What is important to you for the future?
Healthcare and professional support	Clinical support: Healthcare staff who care, feeling well looked after, having treatments explained (11,9) Good access: Being able to see a doctor or nurse when necessary (9,7) Medication: The right medication when needed, feeling empowered to use it (6,9) Feeling safe: Receiving support to feel safe (4,0) Great staff: Terrific, supportive and kind staff (4,0) Listening and understanding practitioners: Someone to talk to who listens and understands (4,0) Complementary support: Exercise referral was helpful (1,0) Feeling in control: Feeling empowered and able to use the system(0,2)	I don’t think the right hand knows what the left hand is doing^a^: Mistakes, delays, duplication, can’t get what one needs, lack of information (12,3) I can’t get appointments when I want them^a^: Frustration with availability and booking systems (4,3) I’m not treated as a person^a^: Feeling pre-judged, not having my COPD treated, insensitive communication (4,2) I’m confused by conflicted advice: Being told different things by different people, not being kept up to date (3,0) I’m angry when there are errors: Receiving wrong information, drugs or treatment (3,0) I feel rushed: Not enough time with the doctor (2,0) I was ill-informed: Not informed about side-effects or condition (2,1) I haven’t had enough physiotherapy: Wanted more support after discharge or rehab (2,1) Access to healthcare staff: Not able to see a GP who knows me (0,3) Other: Poor communication, long waits in the waiting room, dislike of group exercise, second rehab not as good as first, not enough is known about COPD, hospital not interested enough in COPD (0,6)	I want help in all respects from public services: Help with housing, benefits, support for family members, information about support groups (12,4) I would like good clinical and professional support from professionals with the right knowledge: More professional advice and understanding, honest communication, prompt medication, good communication among HCPs (6,6) I want to be seen by my own doctor when I need it, on time: Being seen by the same doctor who knows me and on time (3,0) I would like more organised exercise: Exercise classes (3,0) Look at me when I talk to you: Look at the patient when talking to them (0,2)

^a^Themes voted as highest priority themes in the ‘what’s not working’ category

**Table 2:** Living with COPD themes generated from the one-to-one responses with a short description of each theme [numbers of statements contributing to each theme are shown in brackets as (high priority, lower priority) statements]

	What is working well?	What is not working well?	What is important to you for the future?
Living with COPD	Looking after myself and others: Enjoying pastimes, domestic jobs, pushing oneself to do things, planning ahead, giving up smoking (13,0) Keeping independent/busy/well: Working, travelling, shopping, domestic jobs, hobbies, exercising, being able to do what one wants (12,5) Family and friends support: Support for day-to day activities, support for family members (11,6) Communication: Keeping in touch through computer, help with dyslexia (2,0) Transport: Good public transport, scooter for mobility (2,0) Location: Living close to town (1,0) Other: You’ve got to work at this illness, it’s depressing (0,1)	I can’t do what I want to do^a^: Cannot do hobbies, daily tasks, social activities, difficult to plan, restricted choice (16,10) I live in fear: Fear of breathlessness and the future (6,4) I don’t have enough energy: No energy for daily activities (6,0) I’m anxious and depressed^a^: Low mood, pain, stress, worry (5,0) I can’t eat well^a^: Poor appetite, restricted diet (4,0) I feel like I’m begging: Lack of understanding at the Job Centre (3,1) I’ve lost my mojo: Lack of motivation for exercise/stopping smoking (3,1) I don’t have enough money: Cannot heat the house or afford the bus (2,1) I feel like a burden: Have to rely on people, ask for more dressings (2,0) I am lonely: Want to get out more, not natural to live alone (2,0) I keep forgetting things: Forgetting things (1,0) Other: I think it’s the smoking that’s making me worse (0,1)	I want to stay independent for ever: Being able to enjoy hobbies, look after others, do daily tasks, stay out of hospital (10,6) I want to stay in my own home: Manage in one’s own home for as long as possible (9,3) I want to be mobile: Be able to walk, move around the house, travel abroad (7,0) I would like to stay as healthy as possible to achieve my aspirations: Important to keep well, be able to work and see family (6,1) I need help to stay confident: Finding a reason to do things, meeting people (6,0) I value family support: Having family around (3,0) I want to be able to maintain my social network: Staying in touch with friends (2,6) I want to do the things I enjoy: Doing the things I enjoy (0,3) I want my family to be looked after: Security and care for dependents (0,2)

^a^Themes voted as highest priority themes in the ‘what’s not working’ category

### What is working well?

Healthcare and Professional Support themes included *Clinical support* and *Good access*. (My care team are good and look after me well; I can get a doctor’s appointment if I need one.) There was also good access to *Medication* with information about how to use emergency medication packs. Participants reported *Feeling safe*. There were *Great staff* and *Listening and understanding practitioners*. (I have complete faith in my GP; I have a doctor who listens, advises—total support.) Exercise referral was helpful as *Complementary support*.

Living with COPD themes included *Looking after myself and others* even though it could be difficult (you’ve got to push yourself to do things—don’t just sit there feeling sorry for yourself; being able to cook for all my family on a Sunday lunchtime) and *Keeping independent, busy and well* (being able to do whatever I want to during the day). These themes were about being able to do domestic jobs, enjoy hobbies and maintain a sense of normality. There was good *Family and friends support* in terms of social interaction, being looked after, and having help to do things (I couldn’t cope without them) *Communication* was helped by using computers and getting help with dyslexia. *Transport* was provided through public transport and owning a scooter and one statement described a good *Location*, living near town.

### What is not working well?

Three Healthcare and Professional Support themes were included in the six highest priorities as voted by participants. Firstly, ***I don’t think the right hand knows what the left hand is doing*** represented frustration with the way that some areas of the health service worked. There was exasperation, for example with cancelled appointments and waiting times (I’m still waiting [for my letter] 6 months later) and the timing of appointments (I want to go to the doctors when I need to, not be called to be checked at other times.) Secondly, ***I can’t get appointments when I want them*** represented frustration with availability and booking systems (I phone at 8.30am and the GP is engaged. I get through 10 min later and all the appointments are gone.) Thirdly, ***I’m not treated as a person*** is about not being seen as an individual or being pre-judged because of breathlessness. (I get COPD treated, not my COPD; look at me not the computer when I come to see you—I want you to listen.)

Other themes reflected frustrations including *I’m confused by conflicting advice* from different people; *I’m angry when there are errors* (I felt angry when a nurse made a mistake—it made the results wrong and I was given the wrong information. They didn’t say sorry)*; I feel rushed during appointments*; *I was ill-informed* (I would have liked my condition explained more when I was diagnosed.) Pulmonary rehabilitation was praised but there was a theme around *I haven’t had enough physiotherapy*. (I wasn’t offered physio…..when I was discharged and I think it would have helped.)

Three Living with COPD themes were included in the six highest priorities voted for by participants. Firstly, ***I can’t do what I want to do*** related to how COPD limited people’s abilities (my condition means it’s often not easy to plan social events and holidays and it’s often a waste of time.) This included holidays and hobbies (not being able to go fishing which I enjoy.) Everyday tasks such as housework or exercise became difficult and people’s choices were restricted (I can’t do what I used to do—it’s frustrating and depressing.) Secondly, ***I’m anxious and depressed*** meant low mood and black days. (I feel like I am in a shell and cannot get out of it.) Thirdly, ***I can’t eat well*** was due to poor appetite and being too breathless to eat, feeling unwell, having a restricted diet and poor teeth and dentures.

Other themes included *I live in fear* with anxiety about breathing. (The breathlessness when I’m really unwell makes everything such an effort and it’s scary.) This could narrow someone’s life. (I put off doing things because of fear of attacks of breathlessness.) The theme *I don’t have enough energy* was about feeling breathless and tired and *I’ve lost my mojo* was about a lack of motivation for exercise and stopping smoking. (I’ve stopped exercises since rehab; I find it impossible to motivate myself at home.) *I feel like I’m begging* concerned not being understood by the Job Centre which was a source of worry. (The Job Centre and DWP [Department for Work and Pensions] are making me ill with worry and what they are physically making me do; the lack of understanding and communication at the Job Centre is degrading.) *I don’t have enough money* referred to not turning the heating on in winter and not going out because the bus fare cost too much. *I feel like a burden* was due to having to rely on other people and *I am lonely* was due to living alone and not having anywhere to go. (I’d like to get out more and have somewhere to go—I’m desperate for a club.)

### What is important to you for the future?

Healthcare and Professional Support themes included *I want help in all respects from public services* which ranged from wanting a flat and not a caravan because the cold affects breathing, to wanting to know that family will be supported and having help to remain independent and active. (I want to get the PIP [Personal Independence Payment] and blue badge because it will allow me to get out of the house and not be completely housebound.) *I would like good clinical and professional support from professionals with the right knowledge* was about healthcare support including receiving medication promptly, seeing staff who understand COPD and are honest so that the patient can plan their life and the way in which they access healthcare. (I prefer to be called to see how I am instead of a review, it would save everyone time and money.) The theme *I want to be seen by my own doctor when I need it, on time* was about having a doctor who knows the patient and *I would like more organised exercise* was about formal support following pulmonary rehabilitation. Two responses that were not allocated to the workshop themes related to the need for healthcare staff to look at the patient when talking to them, which was also indicated in the ‘what’s not working well’ category.

Living with COPD themes included *I want to stay independent forever* which referred to not being a burden and doing things for oneself (staying as well as possible so that it’s less likely that I will have to be looked after by others.) *I want to stay in my own home* was linked to maintaining independence. (It’s important to be able to manage by myself at home; to stay in my own home as long as possible, I do not want to go into a home.) *I want to be mobile* meant being able to travel to see relatives as well as being mobile around the house. (I need to be able to carry on being able to walk to the stairs so I can get to the loo.) *I would like to stay as healthy as possible to achieve my aspirations* included continuing to work, seeing grandchildren grow up and living as long as possible. (Giving up smoking is the most important thing to me and will make the difference between seeing and not seeing Christmas.) *I need help to stay confident* included finding an interest and meeting other people. (I want to find a way of motivating myself to be independent; it’s important to stay happy and to be able to have a giggle together.) *I value family support* and *I want to be able to maintain my social network* represented the importance of family and friends. (I would like to recapture my friendships with those I have lost.)

### Solutions generated by participants

**Fig. 1:**
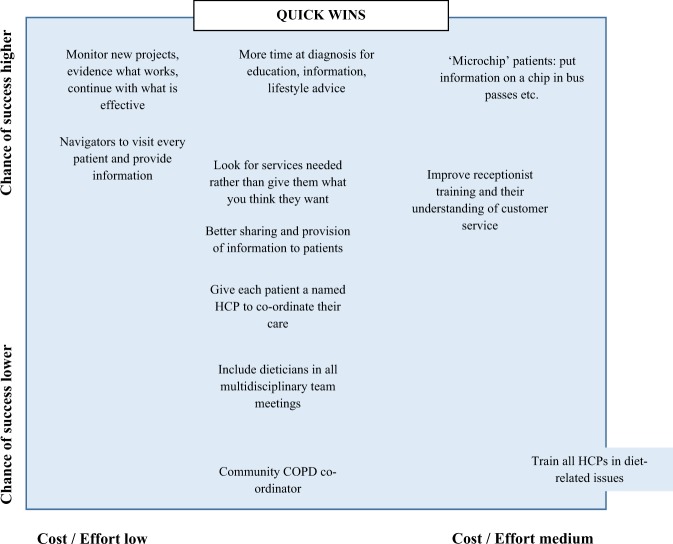
Solution grid showing quick wins

**Fig. 2:**
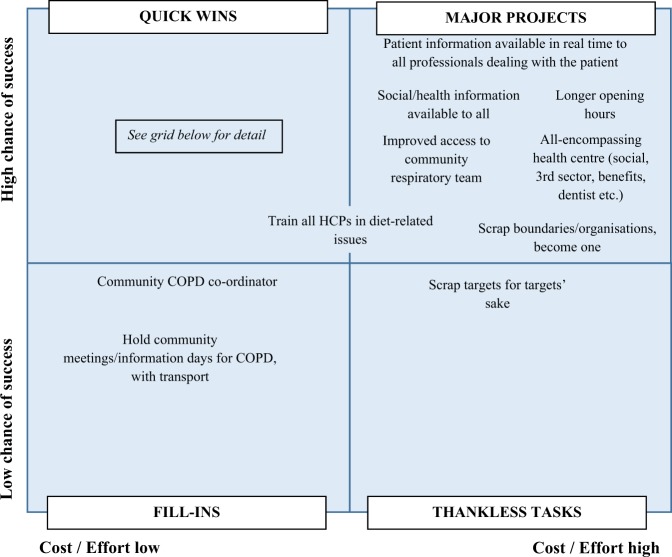
Solution grid showing major projects, fill-ins and thankless tasks

Participants generated nineteen solutions to address the six high priority themes under the heading ‘what’s not working’ (Figs. 1 and 2). Eleven were categorised as ‘quick wins’, i.e. they were expected to have a high chance of success for relatively less cost and effort. Six ‘major projects’ were considered to have a high chance of success but would require more cost and effort. Two solutions were lower priority because they were less likely to successfully address the issues and in one case (scrapping targets for targets’ sake) had a high implementation cost. ‘Major projects’ were more focused on healthcare systems whereas ‘quick wins’ related mostly to individual support and patient-healthcare provider relationships.

### Evaluation of professionals’ learning

Seven professionals completed evaluation interviews (three commissioners, two clinicians, two-third sector). Two key themes described how professionals’ understanding of patient needs had been deepened and broadened: *Gaining new insights* and *First-hand knowledge—witnessing the lived experience of COPD*.

Gaining new insights involved learning novel information and realising the salience of issues. Some professionals expressed surprise at issues that patients raised, some of which they had not been previously aware or of which they had not appreciated the importance. This included the mental health aspects of COPD and why patients cannot always follow the medically advised course of action because of practical or physical limitations.

*“….I was very surprised about what came out actually….you know, for example ‘I’m too breathless* to *eat’ was not something that had been particularly high on my register before…I didn’t appreciate what was important to patients and the outcomes in many ways were generic actually.” [GP 11]*

*“…I suppose it has made me think more about…what might be influencing a patient’s decision not to do something, well why a patient can’t do X, Y, Z which is supposed to be the best medical thing to do.” [GP 11]*

*“I think the main thing was the mental health aspects…Loneliness, depression, isolation…It’s quite different hearing it from other people to reading it on a page…” [Commissioner 15]*

First-hand knowledge—witnessing the lived experience of COPD was gained by spending time interacting with patients and carers. Professionals could not only *hear* the issues patients had and the changes they wanted but could also *see* the reality of their lives, how they functioned and the issues they had to deal with. The impact of this was emotional and humbling.

*“I could see how much… a lot of them were struggling, definitely and that sort of brought home to me the struggles that people with COPD have literally just walking…So yeah, I suppose it enlightened me a bit more to their [struggles], to be honest.” [Commissioner 16]*

*“…[I saw] some terribly humbling things around not being able to eat and breathe at the same time…shouldn’t be surprised but I was…having that real glimpse of their lives, it was really important for me…it was that sort of reality check I think…their lives were clearly changed and influenced…” [Third sector professional 8]*

## Discussion

We adapted the WTfC process to identify what matters to people living with COPD and to use this information to inform the planning of COPD services. In this paper we report what was learned about what matters to people living with COPD.

Where healthcare and social support worked well for people they felt independent and enjoyed support from family and friends with good access to amenities. Where this support was not working well participants reported difficulties with some aspects of the healthcare system. Difficulties that were voted as the highest priorities and that really mattered to patients included feeling that ‘the right hand does not know what the left hand is doing’, that appointments were not available when they wanted them and that they were not always treated as a person. Other high priority difficulties included managing the impact of COPD on patients’ lives: not being able to do what you want to do, feeling anxious and depressed and not being able to eat well really mattered to patients. Looking to the future, it was important to people to have continued healthcare and social support, in particular to be treated with respect, to be able to stay healthy, confident and independent and to maintain social and family contact. The solutions generated suggested quick wins could be had from lower effort interventions that impact patients’ ability to live well with COPD and to have effective relationships with healthcare providers.

For the professionals who took part, WTfC enabled them to gain new understanding and appreciation of the difficulties that patients faced and to learn first-hand about the realities of patients’ lives.

The desire to be independent, to do domestic tasks and hobbies, to maintain control, to be confident, healthy and maintain social activities and relationships with family and friends cut across what was working well, not working well and what was important for the future. Some participants felt independent and could do chores and hobbies but others felt restricted and felt like a burden to family and friends. Mobility was important to many. Williams et al. (2007)^5^ found that people with COPD had a strong desire to engage with physical and social activities, such as walking, domestic tasks and socialising. These were important beyond their functional purpose because they facilitated social integration, role fulfilment and independence, and provided a sense of normality and purpose. Driving in particular provides freedom and improved quality of life.^5,19^ In a meta-ethnography of how people with COPD value different activities Lindenmeyer et al.^20^ found that basic activities such as getting dressed were an important part of trying to live a normal life; doing housework and gardening contributed to feeling useful and not being a burden to others; social activities fostered a sense of belonging even though for many people this was restricted to immediate family. Social interaction enables people to ‘participate’ rather than just ‘do’^5^ while quality of life, freedom and independence have been shown to be dependent on the practical and emotional support from family relationships.^21^ Loss of activities can threaten a person’s sense of self and continuity between past, present and future^22^ and in this way chronic illness has been viewed as a biographical disruption.^23^ People want to be seen as essentially the same person in spite of their illness and activities of daily living are meaningful because they contribute to a sense of continuity of self, enabling enjoyment and a sense of connectedness and effectiveness.^20^

Social isolation and loss of control may also impact mental health. Anxiety and depression were important issues for our participants and are associated with more impaired quality of life and reduced adherence to treatment in COPD.^24,25^ Such feelings may be compounded by fear of what the future holds.^21,26^ In particular, women with COPD experience higher levels of anxiety and depression than men.^27–33^ There is some evidence that women are more likely to use emotion-focused coping strategies (characterised by anxiety and depression), while men are more likely to use problem-focused strategies.^34^

Being able to able to eat well was important for our participants. Difficulty with meal-related situations has been reported in people with COPD, with impacts on nutritional intake due to breathlessness, social impact of eating, altered appetite and ability to get food.^3^ Some of the health professionals who took part in WTfC had not appreciated the extent of difficulty experienced by some patients and were surprised at witnessing this in the workshops. Whilst loss of mobility, independence and social connectedness occur more generally among people living with long term conditions and multimorbidity^35^ the specific difficulty that COPD causes with respect to eating is not a generic issue and may require dedicated support.

Not being treated as a person when interacting with healthcare professionals and feeling that the disease was treated rather than the individual was an important theme. Svedsater et al.^36^ reported that patients with COPD and asthma focused on life impact rather than symptoms, e.g. being able to do everyday activities, the emotional impact of embarrassment due to coughing or wheezing, fear, sadness, not being able to do things and feeling useless. Clinical experts on the other hand focused on symptoms rather than the impact of symptoms on patients’ lives. In their study clinicians were aware that many areas of patients’ lives were affected but did not discuss the impact of symptoms on physical abilities to the same degree as did patients. Indeed, clinical expert assessments and patient-reported outcomes often differ in COPD^37^ and there can be tension between the medical profession’s focus on function, e.g. distance walked, and people’s experience of what is important, e.g. looking after children.^38^

Professionals in our study were enlightened by witnessing the impact of COPD on people’s lives in close contact. Some were surprised by what they learned, which suggests that professionals do not necessarily appreciate what is important to patients. WTfC deepened their understanding of patients’ needs through witnessing patients during shared activities and hearing first-hand accounts. Through witnessing this lived experience of people with COPD, professionals gained insight consistent with wisdom as opposed to knowledge, where wisdom is concerned with the understanding of value and knowledge relates to technical competency.^39^ Such wisdom can complement and maximise the effectiveness of technical interventions enabled by modern medicine and mitigate the immense power that clinicians can have over patients’ treatments and choices.^40^

Two aspects of healthcare organisation were perceived as not working by some patients: service co-ordination and access to appointments. In the UK, NHS (National Health Service) England has recognised the need to optimise services for people with COPD and has published the ‘NHS RightCare Pathway: COPD’.^41^ This pathway provides a case for change, together with resources, to support improvement efforts within local health economies that aim to address variation and improve population health. Dissatisfaction with access to GP appointments in the NHS is an issue experienced beyond the COPD population with satisfaction declining between 2010 and 2016.^42^

Our findings highlight aspects of the COPD experience and of healthcare that matter most to patients. The outputs from WTfC emphasise the importance of individualised care and flexible approaches so that people are seen as individuals and can be supported to be independent in how they live their lives. This goes beyond medical treatment and represents a shift from doctor-driven care towards more patient-centred integrated care with active involvement of the patient.^43^ Being treated as a person mattered very much to participants. In a recent systematic review increased continuity of care by doctors was associated with lower mortality and this supported previous findings of an association between continuity of care in general practice and greater patient satisfaction, improved health promotion, increased adherence to medication, and reduced hospital use.^44^ The authors suggested that where continuity of care takes place patients may perceive the doctor to be more responsive which leads them to disclose more such that medical management is then more likely to be tailored to their needs as a person. More individualised care and self-management support can be provided through pulmonary rehabilitation for people with COPD but access is not universal in the UK and needs to be improved.^45^ Many solutions proposed in this study addressed the need for patient-centred and personalised care and the majority were deemed to be ‘quick wins’, e.g. navigators to visit patients, more time at diagnosis and a named HCP to co-ordinate care.

Support for emotional needs may be particularly important for women living with COPD who may benefit more from programmes that include emotional support and social interaction,^46^ although more research is needed into the differential support needs of men and women.^47^ Support for diet and nutrition is important and our outputs suggest that focused interventions to train dieticians in COPD related issues would be welcome and beneficial.

Addressing what matters to people with COPD requires action at both individual and system level. Solutions that were categorised as ‘major projects’ focused more on integration of services and information and on access. In the UK there is a call to base core national measures on health and well-being outcomes that are most important to people, such as independence, quality of life and feeling supported to achieve these, and to prioritise support for commissioners to build skills, knowledge and confidence to commission for the outcomes that people value.^15^ Principles of personalised care, integrated care systems and improved access to care aided by technology are now enshrined as objectives in a 10 year plan for the National Health Service in the UK.^48^ Responding to issues of GP access and co-ordination of services will require policy and funding solutions to support any actions that individual practices or local health economies can take.^42^ Some solutions will be more readily implementable than others. The solutions generated by WTfC represent ways of achieving these aims, and identify ‘quick wins’ which are relatively easier to implement.

We aimed to be inclusive of all patients and there was a good gender balance. One-to-one interviews held in the patients’ homes were time-consuming but ensured that the views of those who do not typically participate in consultation exercises were included. Statements from all interviews were taken to the workshops and thus the voices of those patients who chose not to attend were still heard. Patients attending the workshops may not have been representative, as there was a disproportionate number of Breathe Easy members who may represent a group more engaged with healthcare matters but other voices were included in the workshops through their statements. It is inevitable that some people contribute more in workshops than others. A larger number of participants for the interviews and workshops may have provided greater variety of expressions but would have been unwieldy. Transferability of the solutions to other health economies will depend on similarities and differences in context but the WTfC process can be applied to generate local context specific solutions.

In conclusion, a range of issues mattered to people living with COPD, encompassing not just health-related issues but also meaning, purpose and relationships, as well as ease of access to health services and the importance of being treated as a person. The findings add support to previous work in this area and underline the importance of a personalised, patient-centred, holistic approach to healthcare for people with COPD. It is suggested that improvements at both patient-clinician level and system level will be necessary to achieve this.

## Methods

### Participants and recruitment

We used a purposive approach to identify people with COPD to include a range of disease severity and social circumstances, including travellers. We recruited from the COPD list of a local general practice, the register of a local pulmonary rehabilitation service, secondary care lists in a local acute hospital, a patient support group (Breathe Easy) and a local self-management support group. People who were not active in patient liaison activities were targeted. Through local contacts and networks we invited professionals involved in care or support of people living with COPD, including clinicians, managers, commissioners and individuals in third sector and public sector organisations.

We aimed to recruit 40 patients for one-to-one discussions and a balanced representation of patients, carers and professionals to attend two subsequent workshops. All patients and their carers who took part in one-to-ones were invited to attend the workshops.

Recruitment, one-to-one discussions and workshops took place between June and September 2013.

### Working Together for Change process

Details of the WTfC process have been published^18^ and an overview is attached in Supplementary Table 2. Briefly, WTfC follows a six-stage framework in which information from person-centred reviews is analysed in two one-day workshops using co-production principles and quality improvement techniques.^17^ Person-centred reviews are not a routine feature of COPD care and so we adapted this stage and conducted dedicated one-to-one discussions in participants’ homes. These were conducted by members of the project team (KH, TW).

In the one-to-one discussions participants were asked three specific questions in relation to their COPD care and living with COPD: what is working well, what is not working well and what is important to you for the future? The interviewer wrote down their responses and asked the participant to indicate one or two responses to each question that were most important to them.

These most important responses were then taken to the workshops to be analysed by patients, carers and professionals working collaboratively. Participants themed the responses by grouping similar types of responses together and then naming the theme. They then voted for what they thought were the highest priority themes under the heading ‘what’s not working’. Following the workshops, FE and ML allocated the responses from the one-to-ones which had not been analysed in the workshops to the workshop-derived themes where possible, otherwise noting them separately. This paper reports the themes that resulted from this stage of the process.

The next stage of analysis within the workshops was to explore root causes of the six highest priority themes under the heading ‘what’s not working’ and then vote on the main root cause of each (Supplementary Figures 1 and 2). Success statements were formulated to describe what success would look like from the perspective of patients, commissioners, healthcare professionals and the third sector if the main root causes were addressed (Supplementary Table 3). Finally, solutions were generated and ranked along two dimensions according to the cost and effort involved and the likelihood of success. These solutions are reported in this paper.

### Workshop evaluation

Following the workshops semi-structured interviews were conducted by FE, PF, AG, KH and TW with patients, carers and professionals to explore their experiences and satisfaction with the workshop process and, for professionals, any insights they gained into patients’ needs. Interviewees represented a range of circumstances and roles. Interviews with patients and carers were conducted in their homes and with professionals at their place of work. Average duration was 36.5 min (range 14–77 min). They were audio-recorded and transcribed. Transcribed interviews were imported into NVivo 10 software and analysed thematically.^49^ A deductive coding frame was defined from the interview topic areas by FE. All transcripts were read FE and SW who independently identified emergent codes which were then reviewed and agreed by FE and SW and incorporated into the coding frame. All transcripts were coded for all codes by SW. Codes were checked for duplication and redundancy and organised into themes and sub-themes by SW. FE reviewed the themes and sub-themes and SW and FE agreed the final themes. Themes relating to the workshop process have already been published.^18^ In this paper we describe the themes that related to new insights gained by the professionals into patients’ needs.

The project was designated as a service evaluation at Cambridge University Hospitals NHS Foundation Trust (Project Registration No. 2789). Before the first workshop commenced KH and TW took informed written consent from all participants to take part in the evaluation.

### Reporting Summary

Further information on experimental design is available in the Nature Research Reporting Summary linked to this article.

## Supplementary information

Supplemental Material

Reporting Summary

## Data Availability

All of the responses that were written down by the interviewers during the one-to-one discussions are presented in Supplementary Table 4.

## Supplementary information

**Supplementary information** accompanies the paper on the *npj Primary Care Respiratory Medicine* website (10.1038/s41533-019-0124-z).
