# Supplementary material for: What matters to people with COPD: outputs from Working Together for Change
Source: NPJ Prim Care Respir Med. 2019 Apr 12;29:11. doi: 10.1038/s41533-019-0124-z (PMC6461642; doi:10.1038/s41533-019-0124-z)
Supplement: Supplementary file 1 — Supplemental Material [file 41533_2019_124_MOESM1_ESM.pdf]

PATIENTS/CARERS Source of contact	Approached	Participated in one-to-ones	Attended workshops
Breathe Easy support group	10	9	7
GP list	37	25	4
Acute respiratory ward	3	2	0
Pulmonary rehabilitation register	7	7	0
Self-management programme	4	1	0
Occupational Therapist	1	1	0
Invited directly to workshop	-	-	3
Carers	-	-	8
**Total**	**62**	**45**	**22**
**PROFESSIONALS Sector/Organisation**	**Approached**	**Attended day 1**	**Attended day 2**
Acute Trust	5	3	2
Acute Trust / HCP*	4	2	1
Ambulance Service	1	0	0
CCG*	6	1	2
CCG / HCP	1	1	1
Community Trust	2	0	0
Community Trust / HCP	2	1	1
NHS England / HCP	1	0	0
Healthwatch	1	0	0
Housing	2	1	1
Public Health	2	0	0
Public Health / HCP	1	0	0
Multi-agency	3	1	1
Voluntary Sector	11	5	7
**Total**	**42**	**15**	**16**

*Healthcare Professional *Clinical Commissioning Group

Supplementary table 1: Workshop participation

**Stage 1**	**Gather person-centred information from patients: One-to one discussions in the home**<br>Explore with regard to the person's COPD a) what is working in their life, b) what is not working and c) what is important for the future. The patient indicates the two most important statements in each category. Patient and carer are invited to attend the workshops.
**Stage 2**	**Workshop preparation by the project team**<br>Each patient's most important statements are transferred to cards for use in the workshops.
**Stage 3**	**Workshop activity: Cluster the priorities into agreed themes**<br>Workshop participants (patients, carers and professionals) work together with a facilitator to cluster the important statements into themes. This is done for each category: what's working, what's not working and what's important for the future. Each theme is labelled with a first-person statement that best describes the theme, for example "I never have enough time with my GP" and "I feel like I am pre-judged because I am breathless" might be labelled "I am not listened to." Participants are given three sticky dots and vote for what they think are the highest priority themes in the 'not working' category. The six highest priority themes are taken forward to the next stage.
**Stage 4**	**Workshop activity: Analyse the information**<br>Groups of five to six patients, professionals and carers analyse the root causes of the six priority 'not working' themes. The group asks the question 'why' several times to generate as many root causes as possible. Each group identifies their five most important root causes, pins them on a board and everyone dot votes for what they think are the most important root causes. Success statements are formulated to represent what success would look like if each root cause were addressed. Success statements are considered from the point of view of service users, commissioners, clinicians and practitioners and the third sector, and generated under the headings 'radical', 'traditional' and 'different' to encourage creativity whilst recognising the best of what already exists.
**Stage 5**	**Workshop activity: Action plan**<br>Participants discuss what could be done differently to generate the successful outcomes. Solutions are ranked according to the risk/benefit involved and ease of implementation, resulting in four categories of actions: major projects, quick wins, fill-ins and thankless tasks. For each action the group discuss and agree: where do you want to be in six months' time, what needs to happen first, how will you ensure co-production principles persist in working towards this action?
**Stage 6**	**Post-workshop activity by the project team: Share the information**<br>Outputs from the process are summarised in a report by the project team and circulated to commissioners for their consideration.

Supplementary table 2: Overview of the WTfC process applied in COPD context

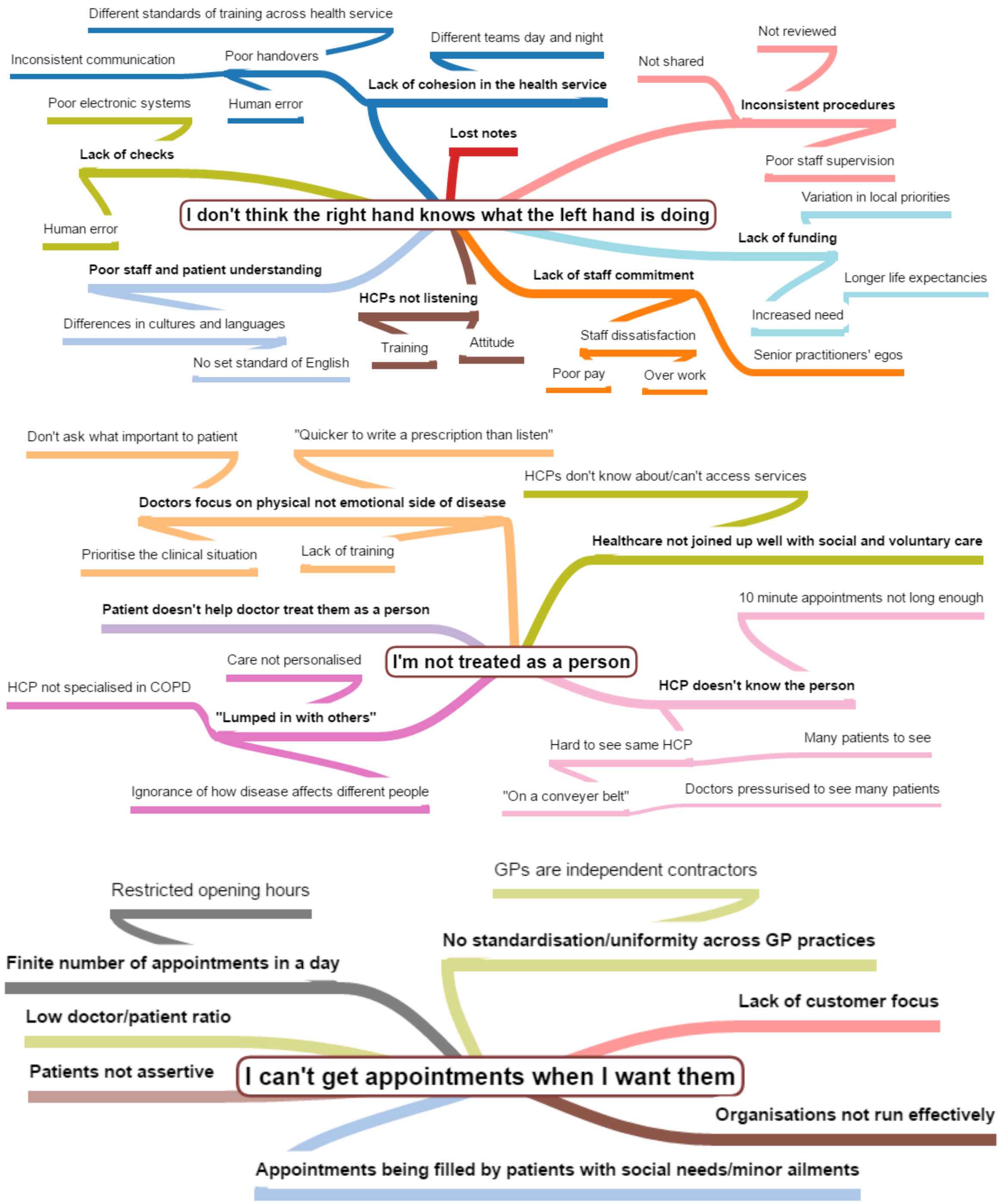

Supplementary figure 1: Root causes of the prioritised 'healthcare and professional support' themes in the 'What's not working category'.

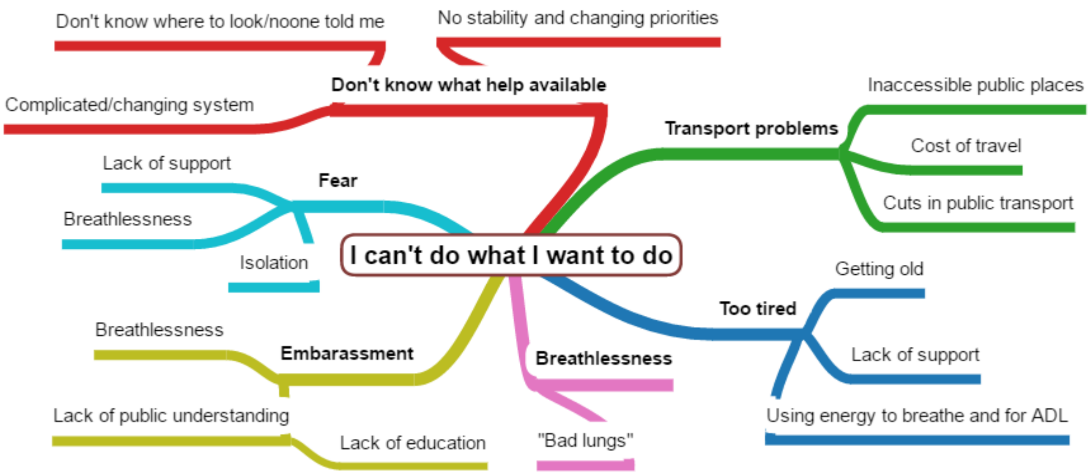

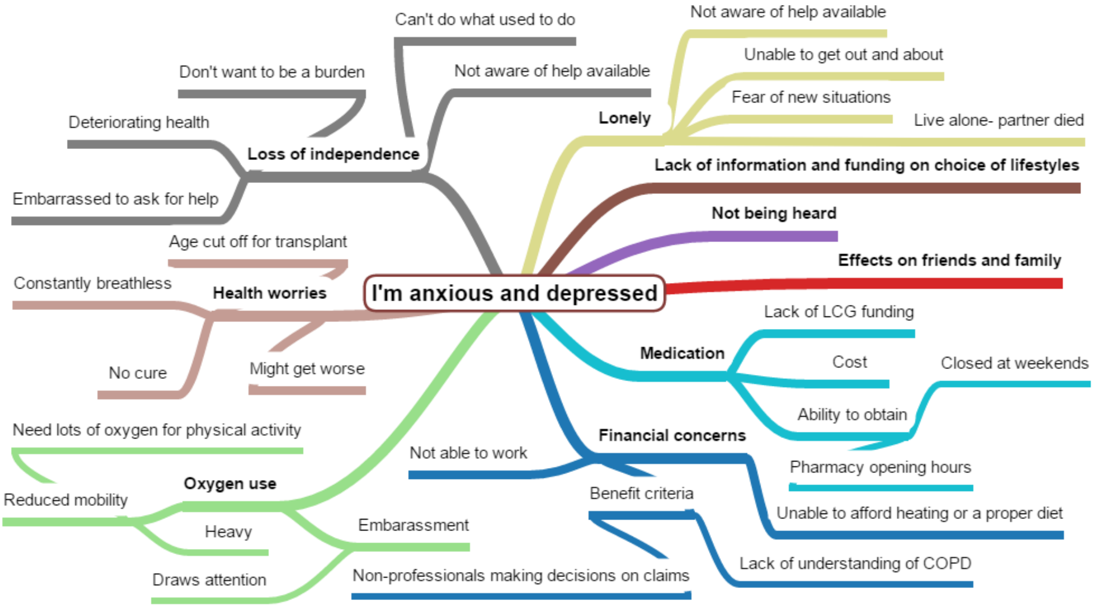

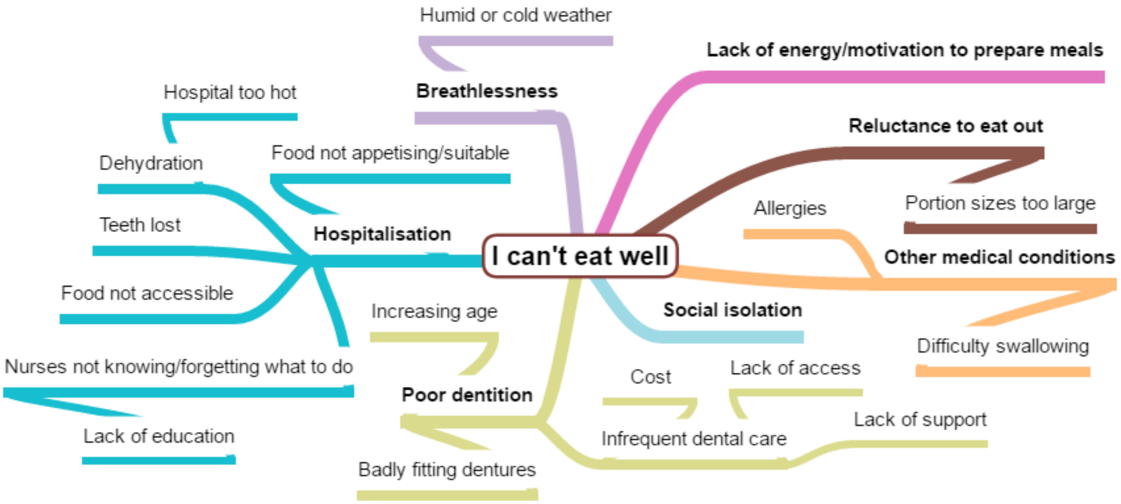

Supplementary figure 2: Root causes of the prioritised 'Living with COPD' themes in the 'What's not working category'.

		Success statements			
**What's not working priority themes**	**Most important root cause**	**From service user perspective**	**From HCP perspective**	**From commissioner perspective**	**From third sector perspective**
**I don't think the right hand knows what the left hand is doing**	Inconsistent procedures across organisations and departments with no checks and balances	Everything runs smoothly with no hiccups and I am satisfied	The system works well because I know what is expected of me. It's the same everywhere I work and it tells me if I make a mistake	We commission services that have consistent procedures and adequate checks and balances in place	HCPs refer to and use our services giving us the right information at the right time
**I can't get appointments when I want them**	Doctor/patient ratio	I can get an appointment when I want one	We can see patients when they want to see us	We would meet patients' access needs	We would have sufficient capacity to support all individual needs
**I'm not treated as a person**	Health isn't joined enough to social/3rd sector care so that people don't know about/can't access the right services	I know where to go to and how to access the help and advice I need	We can easily access help and advice for people when they need it	We use a pooled budget to give our population a better quality of life and value for money	We feel our contribution has equal value and is respected and included
**I can't do what I want to do**	Lack of knowledge of organisations and services, support groups available and how to access them	I know exactly what help is out there and how to access it	We have an easily accessible, one stop, multi-organisational network of support available to all patients	We support and facilitate the integrated approach to care/support provided by the multi-organisational network	We are a fully integrated part of the one stop, multi-organisational network easily accessible to patients
**I'm anxious and depressed**	Lack of information of choice of lifestyles	I have access to information that enables me to choose the lifestyle I want	We involve other agencies to enable patients to change their lifestyles	We have commissioned the services that enable the patient to live the lifestyle they choose	We have enabled the patient to live the lifestyle of their choice
**I can't eat well**	I find it difficult to eat and breathe at the same time	I can eat normally because my breathing doesn't affect my eating	I have provided techniques/treatment to enable my patient to eat well	I commission the right amount of the right services to enable patients to eat well and remain well and less in need of healthcare services	I have the expertise and resources to support people in eating well

Supplementary table 3: Success statements for the six high priority themes in the 'What's not working' category

The first number in the brackets indicates the responses that were themed in the workshops and the second number indicates the responses that were allocated to themes after the workshops.

What is working well: Healthcare and professional support

Clinical support (11,9)

1. I stopped smoking when I had a new GP, I was looked after and supported through it, I even got a certificate!
2. My care team are good and look after me well
3. I get enough time with my GP - they give me what I need and know me well.
4. Half the battle is having carers that care – I have a personalised care budget that I top up and I chose my care company.
5. I have a good relationship with my GP and nurse practitioner – they really help me because they understand my situation
6. I feel like my GP gives me enough time to tell them everything I need to.
7. My wife had excellent treatment at the hospital; every question I asked was answered well.
8. It's important for me to have everything explained to me so I understand and this has happened every time.
9. The pulmonary rehab course was superb, especially the information and exercises
10. I get help even though I find reading hard.
11. I get my drugs delivered at home which means I don't have to go out

1. The hospital have explained everything well, even a nurse who said she did not have the experience to explain a test result passed me over to someone who could explain better.
2. I can talk to [woman's name], she is not in a rush and I get the time I need.
3. I rely on my annual [hospital] appointment – I like the environment there.
4. I was taken good care of by my consultant.
5. I take my hat off to [hospital], they have been amazing.
6. [Hospital] is good.
7. Pulmonary rehab prepared me for the symptoms I've got to look out for.
8. The rehab lectures were good – nobody had told me before how to bring up phlegm, it was amazing.
9. I have learnt my breathing exercises from rehab and still do them

Good access (9,7)

1. If I say I have a breathing problem I can be seen straight away by my doctor.
2. It's easy to make a doctor's appointment at my surgery
3. I know I can ring [woman's name] in the morning and somebody will be out that day – they're very good the respiratory team.
4. I can see the nurses at my GP practice when I need to
5. I can get a doctor's appointment if I need one.
6. I get to see a GP in a few days
7. I can get an appointment within a week.
8. I get to see my nurse/doctor within a week
9. I can email my consultant and contact him when I need to that gives me confidence.

1. The clinic on Saturday morning has been amazing, it beats sitting in a bust clinic in the week.
2. I can see the same doctor if I need to.
3. I can get an appointment within 48 hours.
4. If it's not urgent I can be seen within a week no problem
5. It's on my GP records that I need to be seen when I call so I do not have any problems getting an appointment if I need one.
6. We have a great hospital; I have been treated well, on time and always get a response when I call.
7. I feel like I have enough time with my GP even if it's a doctor I have never seen before

Medication (6,9)

1. Without my inhalers life would be more of a struggle
2. Getting the right medication and on time
3. I get my medication when I need it.
4. The medical attention and medications are very important and very good
5. I know when I have a chest infection I can use my emergency antibiotics
6. I am empowered; I feel educated enough to know how to use my emergency pack when I need to.

1. I get my repeat prescriptions on time.
2. I can get my medication easily; it all works well for me.
3. I like the fact that my repeat prescriptions are delivered
4. I have my emergency packs at home I can use when I need them.
5. I have an emergency pack at home which makes me feel secure.
6. I have an emergency pack

7. *I wouldn't be without my nipi now – it really helps.*
8. *My nebuliser is the best £65 I've spent in my life*
9. *I know what my SATs are, I can measure my own levels and I have enough money to buy my own meter.*

Feeling safe (4,0)

1. My telehealth team are on the ball; it makes me feel safe knowing that I am monitored daily.
2. I have good support from my consultants, doctors, family and friends – it makes me feel safe
3. I feel safe as I have my emergency antibiotics and steroids at home.
4. Since pulmonary rehabilitation I feel less worried about being short of breath

Great staff (4,0)

1. I have complete faith in my GP, they are supportive and kind.
2. The enthusiasm the staff have for getting to the bottom of my illness is applaudable and terrific
3. I click with my support worker; I would find life very difficult without him.
4. My GP and staff at [hospital] are wonderful and staff on [ward] are brilliant.

Listening and understanding practitioners (4,0)

1. Having a GP that understands COPD
2. I have a doctor who listens, advises – total support
3. Continuing to have a GP and practice nurse that I can talk to
4. I have an excellent GP practice that is very understanding and works well together

Complimentary support (1,0)

1. The exercise referral to the gym has been really helpful.

Feeling in control (0,2)

1. *The medical system works perfectly for me; I am empowered and have control.*
2. *I have learnt how to get what I want from the system.*

What is working well: Living with COPD

Looking after myself and others (13,0)

1. I enjoy long green bowling with my husband and keeping fit
2. Being able to go for a walk
3. We can still go out and see friends and family and for lunch
4. Being able to do my housework and cleaning
5. Being able to do my cooking and washing up
6. Being able to cook for all my family on a Sunday lunchtime
7. You've got to push yourself to do things – don't just sit there feeling sorry for yourself.
8. It's important for me to have a go and not give up.
9. Planning everyday tasks ahead helps me manage better and keeps me more independent
10. I can plan more easily because I have looked after my money
11. Knowledge – you have to judge yourself and you've got to know about your illness
12. I've given up smoking and I'm glad – I'm a non-smoker for ever now
13. Feeling ok, not breathless

Keeping independent/busy/well (12,5)

1. I can still work
2. We are still able to travel
3. Going shopping every Wednesday afternoon with my daughter
4. Being able to clean my flat
5. Being able to do my garden, which I enjoy
6. Maintain my exercise class to help with continued mobility
7. Being able to breathe ok and have the energy to do things around the house
8. I love doing crosswords and watching Animal Planet – they keep my brain active
9. I will pick up on my embroidery again during the winter.
10. When I'm well a multitude of doors open up for me – I can do much more
11. Being able to do whatever I want to do during the day
12. Accepting my condition and living for the moment.

1. *If I don't exercise I get pins and needles, I must get up and walk once a day.*
2. *I can do all my housework myself.*

3. *I can get the bed made – it takes time, but I can do it.*
4. *I like where I live, I can mix with others but still have some independence.*
5. *It's important to be able to choose the support I need as I need it.*

Family and friends support (11,6)

1. Friends and family – I couldn't cope without them.
2. My brother's help with my weekly shopping is important
3. My husband's/wife's support to be fitter is really important for both of us – we support each other
4. I am looked after by my family
5. I have good support around me
6. Continued support from my husband and others who help, like my cleaner
7. Having unconditional support from my wife and being together she knows what I need
8. My friends and son help me out and give me a life
9. Keeping a balance of approaches to my COPD with my wife well enough to keep things going
10. I can get out on days and holidays which gets me out of the house and makes a change
11. My daughter who has special needs goes to college and is well supported.

1. *I have a friend who helps me to fill in any forms I need to.*
2. *I have friends to help me to fill in forms as I find it hard.*
3. *I have good support around me.*
4. *We have good support from the telehealth team, friends and family; it gives me the strength to carry on.*
5. *If I want to do something I can go and do it, however I prefer to have somebody with me.*
6. *You have to get a grip and tell yourself you've been here before – if I have somebody else with me it's reassuring.*

Communication (2,0)

1. Contact information through a computer lets me keep in touch when I cannot talk on the phone
2. I am dyslexic and get help when I need it.

Transport (2,0)

1. It's easy for me to get into town, public transport works for me.
2. My scooter gives me more independence to and freedom to do what I want when I want

Location (1,0)

1. I like living close to the action near to town.

Other (0,1)

1. *You've got to work at this illness – it's depressing*

What is not working well: Healthcare and professional support

I don't think the right hand knows what the left hand is doing (12,3)

1. I feel frustrated with the hospital because I am told that I am on the waiting list and the list is 2 months long, I am still waiting.
2. I was told by the medical secretary my letter was waiting to go – I'm still waiting for it six months later
3. Weekend support when involving ambulances and A&E is astronomically expensive and involves long delays in receiving treatment which is very stressful
4. Don't ask me to come back and see you in two weeks if I can't make an appointment there and then.
5. I was cross that [the hospital] cancelled three [clinic] appointments – I was getting worse and they didn't explain.
6. I cannot pre-book appointments with my doctor, I have to call every day
7. I don't want to get a letter about the same thing three times; it makes me feel as if the hospital do not know what they are doing
8. My care team have changed and the hand over was poor so now we are starting from scratch with care plans, I need to be re assessed again, that irritates me.
9. I often run out of drugs because of the way the system works – I want to be able to order them all at the same time
10. It can take a week for me to get my medication; there must be a better way.
11. [The clinic] didn't send me the drugs I needed because the doctor was on holiday
12. I want to go the doctor's when I need to, not be called to be "checked" at other times

1. *The hospital gave me the wrong antibiotics that upset me.*
2. *The GP booklet at [general practice] is out of date. All the GPs are not on there.*
3. *I have to set up a system of letters asking for more drugs so that I get what I need on time*

I can't get appointments when I want them (4,3)

1. Getting a doctor's appointment the day that I need it
2. It's difficult to contact my GP when they don't work full-time if I feel ill in the middle of the week
3. I phone at 8.30 am and the GP is engaged, I get through 10 minutes later and all the appointments are gone!
4. It's difficult to get an appointment when I need to. I get frustrated at not being able to pre-book.

1. I cannot get an appointment the same day
2. It's not easy to see the same doctor
3. I cannot book in advance to see the same doctor

I'm not treated as a person (4,2)

1. He said I could operate but you might die – he could have put it another way.
2. I get COPD treated, not my COPD
3. Look at me, not the computer when I come to see you – I want you to listen
4. If the Dr doesn't understand COPD and I see him about something else he pre judges my breathlessness.

1. My doctor prejudges my condition, that doesn't help
2. When I am not treated as an individual

I'm confused by conflicted advice (3,0)

1. I am frustrated that I am told different things from different people
2. Health professionals having different opinions about treatments makes it difficult for me.
3. I feel frustrated and worried when I am not kept up to date with what's happening, I don't know if I'm coming or going but I don't like to make a fuss.

I'm angry when there are errors (3,0)

1. I was sent home too early from hospital, I was rushed back that night by 999. It was very upsetting, somebody made a mistake.
2. I felt angry when a nurse made a mistake – it made the results wrong and I was given the wrong information, they didn't say sorry.
3. It makes me mad when my GP messes up my drugs

I feel rushed (2,0)

1. I need more than 10 minutes to tell you what's wrong.
2. Sometimes I feel like the doctors do not give me enough time

I was ill informed (2,1)

1. I felt disappointed when I found out myself that there are side effects from some medication I take that I was not told about.
2. I would have liked my condition explained more when I was diagnosed.

1. I don't have much confidence in the nursing team at the practice, they are kind but cannot tell me what I need, I go to [hospital] for that

I haven't had enough physio (2,1)

1. I wasn't offered physiotherapy by [hospital] and [another hospital] when I was discharged and I think it would have helped.
2. It would be good to have the support from physios and amongst patients carry on after rehab

1. I carry too much weight around my tummy and have never been shown how to exercise

Access to health care staff (0,3)

1. I worry as there are so many new doctors, I would prefer to see the same one.
2. My care team are sent to look after others when they should be with me, that makes it dangerous for me.
3. My GP that knows me has left the surgery, I feel like my care will suffer.

Other responses (0,6)

1. I was told by my doctor that one day my wife would come into hospital and not come out again, I didn't want to hear that.
2. Sometimes I wait for 30-40 minutes in the surgery for my appointment that's very frustrating.
3. I don't like being in a group for exercise like PR
4. The first rehab I did with the self-management was brilliant the second one was nowhere near as good, they missed out loads.
5. I don't think enough is known about COPD still
6. [The hospital] is more interested in sleep pattern people than COPD people

What is not working well: Living with COPD

I can't do what I want to do (16,10)

1. Not being able to go fishing which I enjoy doing
2. I'm unhappy that I can't take myself to town anymore
3. I miss work and the social and physical side of it
4. I am so bored because I cannot work – what can I do anyway, I can't read or write
5. I get bored to tears sometimes in my own company.
6. I can't entertain anymore because I find it too stressful
7. My condition means it's often not easy to plan social events and holidays and it's often a waste of time
8. I'd love to go on holiday
9. Not being able to do my exercises
10. I can't get enough exercise because I can't straighten my back and my arms ache on the walker
11. I find it difficult to cope with cooking and housework
12. I don't like having to cancel my plans and not being able to do the things I want to
13. I can't do what I used to do – it's frustrating and depressing.
14. I have to go into town early to get a disabled space as there are not enough. This gives me less choice.
15. COPD means I can't lie on my left hand side when I'm not well.
16. I have had to chase hospital appointments many times, I am promised call backs and it doesn't happen.

1. I get frustrated because I cannot drive.
2. I cannot carry out simple tasks and that's frustrating.
3. I feel a bit pissed off that I cannot do much, I can get depressed.
4. I don't like a dirty kitchen and I'd like to be able to do my own hoovering.
5. I don't like not being able to do as much with my grandchildren
6. I'm looking for work but nothing suits because of my condition
7. COPD is a real life changer – it's very depressing
8. Getting to the doctor's is hard because of my leg pains and breathing.
9. I look for excuses not to meet people
10. I can't get comfy in bed

I live in fear (6,4)

1. I tend to panic about my breathing so quickly but if I have somebody else with me it's reassuring
2. The breathlessness when I'm really unwell makes everything such an effort and it's scary.
3. I put off doing things because of fear of attacks of breathlessness
4. COPD has narrowed my life – we are careful not to put ourselves in a position where I might have an attack in front of other people
5. I have a degree of fear of going a long distance on my own
6. What the future will hold in care and medical support is a worrying factor in this day and age

1. I have a fear of going out on my own.
2. I don't have the time to make decisions, I feel under pressure to make decisions and that's not easy when you are ill.
3. I worry about not getting my drugs.
4. When I exercise my oxygen levels drop and that concerns me

I don't have enough energy (6,0)

1. Not having the energy to change my bed clothes
2. Having no energy and feeling rough
3. Feeling too breathless to go out or get up
4. Having no energy to do anything or go out
5. Feeling too breathless to go out
6. Feeling tired

I'm anxious and depressed (5,0)

1. Feeling very low, I call it my 'black days' when I just want to stay in bed
2. I feel like I am in a shell and I cannot get out of it.
3. I am in pain and frustrated, I cannot get the drugs I got in Germany that take the pain away.
4. Lots of stress effects my health
5. The worst thing is having to go to a new event because it makes me worry

I can't eat well (4,0)

1. My appetite is poor; I don't know what to do about that.

2. My teeth/dentures are no good, I use Fortisip drinks, and I want to be able to eat properly
3. I have a very restricted diet.
4. When I'm feeling unwell I can't eat and I lose weight

I feel like I'm begging (3,1)

1. The lack of understanding and communication at the Job Centre is degrading – they don't understand that I can't work
2. The Job Centre and DWP is making me ill with worry and what they are physically making me do.
3. I am dissatisfied with Job Centre decisions because they expect me to go back to work and don't take account of my illnesses

1. *It was frustrating going through the forms to claim benefits, it's a difficult system*

I've lost my mojo (3,1)

1. I don't always have the motivation to exercise regularly, sometimes I wake up and I feel I can do it, once I have started I can carry on.
2. I have stopped exercises since rehab; I find it impossible to motivate myself at home.
3. It's difficult to break the habit of smoking when you're unwell and don't have anything to do.

1. *It's impossible to find the motivation to exercise.*

I don't have enough money (2,1)

1. A cold house, not being able to turn the heating on in winter
2. It costs too much for me to get on a bus so I stay in

1. *I can't work so I am in debt and I can't get more help, I feel victimised.*

I feel like a burden (2,0)

1. Having to rely on people is a nuisance – I've lost my independence.
2. I feel like a burden when I ask for more medication/dressings

I am lonely (2,0)

1. It's not right or natural to live on my own – I'm lonely
2. I'd like to get out more and have somewhere to go – I'm desperate for a club

I keep forgetting things (1,0)

1. Keep forgetting things!

Other (0,1)

1. *I think it's the smoking that's making me worse*

What is important to you for the future: Healthcare and professional support

I want help in all respects from public services (12,4)

1. I want to be able to fill in my own forms so others don't know all my business
2. I want my daughter to be looked after when I am gone
3. As my condition progresses it's important that my wife gets support if she needs it
4. I would like there to be enough support for me and my wife when my condition worsens
5. I need to carry on getting the help I need, when I need it
6. Being cold effects my breathing, I want to be in a flat and not in a caravan.
7. I'd like a shower fitted to my bathroom as I can't get in and out of the bath anymore
8. It's important to have peace of mind with the job centre because it's no good for my health
9. I want to find out more about the group we heard about at rehab in [town] and Breathe Easy
10. To carry on getting my benefits as they give me independence
11. I want to get the Personal Independence Payment and blue badge because it will allow me to get out of the house and not be completely housebound.
12. NHS funded trips to salt mines, as I've heard these are beneficial to COPD patients

1. *I would like an electric bed that lifts up to help my breathing and my back pain.*
2. *An extra £10 a week so that I can stay warm in the winter.*
3. *I want to stay warm and dry in the winter not damp and cold.*
4. *I need help to fill in forms and to understand the system*

I would like good clinical and professional support from professionals with the right knowledge (6,6)

1. To receive my medication from the hospital pharmacy on discharge promptly. As I often have to wait hours to be

discharged because I haven't received my medication – this is frustrating.

2. I would like non-respiratory staff at my hospital to understand my condition and for the health professionals caring for me to communicate with each other (respiratory and non-respiratory)
3. I prefer to be called to see how I am instead of a review, it would save everyone time and money.
4. It's important to me to continue to have contact and support from the respiratory team
5. I want to get more professional advice and information about my medication and additional treatments
6. Please be honest with me and keep me up to date so I can plan my life.

1. I would like to be in 12 months after rehab to see how I'm doing.
2. I want to be kept an eye on as it makes me feel safe.
3. To have time in appointments and to be listened to.
4. I want to be able to carry with my appointments at [hospital], they are the specialists and have better facilities.
5. It's important for patients to have access to new treatments and know what's available
6. It's important to have my medication on repeat

I want to be seen by my own doctor when I need it, on time (3,0)

1. It's important to be seen on time at the surgery - sometimes I wait for 30 minutes
2. I want to be seen by my GP. They know me best.
3. I want to see the same doctor.

I would like more organised exercise (3,0)

1. I would like more organised exercise classes to attend near home
2. More local exercise classes to attend after pulmonary rehab
3. I would like refresher rehab sessions every 6 weeks please.

Look at me when I talk to you (0,2)

1. Look at me when I talk to you please
2. Look at me and not the computer when you talk to me please

What is important to you for the future: Living with COPD

I want to stay independent for ever (10,6)

1. I want to keep going to the jazz festivals I enjoy.
2. I want to stay as well as possible so I can carry on looking after others
3. Staying as well as possible so it's less likely that I will have to be looked after by others
4. I'd like to be able to look after myself for as long as I can in the future
5. I want to be able to do my own hoovering.
6. I want to stay independent and look after myself with my care team
7. It's important to work around my illness and keep planning, to maintain my independence
8. It's important to me to keep well so that I can maintain the things I'm doing
9. I want to keep well so that I stay out of hospital
10. It's important to be able to get out and do things for myself and be independent.

1. Not to be a burden
2. It's important for me to look after myself as I get older.
3. To stay independent and travel.
4. I have to go into town early to get a disabled space, I would like to choose when I go and go when I feel well enough.
5. I want to stay by myself and make my own decisions.
6. I would like a new pair of legs please.

I want to stay in my own home (9,3)

1. It's important to be able to manage by myself at home
2. I want to stay in my own road as I get older
3. To stay in my own home as long as possible, I do not want to go into a home.
4. It's important to stay independent and in my own home.
5. It's important to stay in my own home as I get older.
6. I'd like to be able to manage at home for as long as we can by ourselves
7. To stay in my home as long as I am not a burden.
8. It's important to be able to stay at home – I don't want to go into a care home.
9. To stay in my own home

1. I want to stay in my own home
2. I want to be able to look after myself in my own home.
3. I want to stay in my own home but not at the expense of others.

I want to be mobile (7,0)

1. I need to be able to carry on being able to walk to the stairs so I can get to the loo.
2. I'd like to be able to walk without any aid.
3. It's important to try and improve my mobility
4. I want to be well enough to see my son in Australia.
5. I want to be able to climb 15 steps of an aircraft so I can travel; it makes me feel like I can get around.
6. It's important to be able to travel abroad in the winter for our health to avoid the British winter
7. Cheaper travel insurance to go on holiday, as I get charged too much money because of my health problems.

I would like to stay as healthy as possible to achieve my aspirations (6,1)

1. It's important to be able to carry on working as long as possible.
2. It's important to see my children and grandchildren grow up
3. Giving up smoking is the most important thing to me and will make the difference between seeing and not seeing Christmas
4. I would like to live as long as possible.
5. It's important to me to keep as well as I am now
6. I hope my lung reduction operation will help

1. I need to work as it's something to do

I need help to stay confident (6,0)

1. I want to find a way of motivating myself to be independent
2. It's important to find an interest to keep you going
3. To have a reason to get up and do things
4. It's important to stay happy and to be able to have a giggle together
5. To be able to meet other people locally who also have COPD
6. I'm frightened of being on my own because I do need support if I become unwell.

I value family support (3,0)

1. It's important to have my family around me.
2. To know my limits with support from my partner
3. Support from my wife

I want to be able to maintain my social network (2,6)

1. I would like to recapture my friendships with those I have lost
2. I want to be able to stay in touch with others

1. It's worth the struggle to meet new people.
2. I want to carry on entertaining.
3. Computer access so that I can keep in touch.
4. My children are important to me - I need them to be close to me.
5. My relationship with my ex-partner is still really important even though we are apart.
6. To carry on supporting Breathe Easy

I want to do the things I enjoy (0,3)

1. I want to enjoy what life I have left
2. I just want to be me
3. I like to eat well.

I want my family to be looked after (0,2)

1. I want to leave some security for my son.
2. We need a rest from caring for our daughter and that she will be looked after well

Supplementary table 4: Responses from one-to-ones
